# Determinants of newborn care utilization in Pakistan: Findings from the Demographic and Health Surveys

**DOI:** 10.12688/f1000research.25700.2

**Published:** 2020-10-21

**Authors:** Sathirakorn Pongpanich, Abdul Ghaffar, Najma Ghaffar, Hafiz Abdul Majid

**Affiliations:** 1 College of Public Health Sciences, Chulalongkorn University, Bangkok, Bangkok, 10330, Thailand; 2 Gynaecology & Obstetrics, Bolan University of Medical and Health sciences, Quetta, Balochistan, 83700, Pakistan; 3 Health Department, Government of Balochistan, Quetta, Balochistan, 83700, Pakistan

**Keywords:** determinants, newborn, postnatal care, utilization, Pakistan demographic health survey

## Abstract

**Background:** Information on determinants of postnatal care is essential for maternal health services, and this information is scarce in Pakistan. This study aimed to determine the factors of newborn postnatal care utilization from the Pakistan Demographic and Health Surveys (PDHS) conducted from 2006–2018.

**Methods:** We analyzed data from three rounds of cross-sectional, nationally representative PDHS 2006–07, 2012–13, and 2017–18. Multivariable logistic regression models were applied to explore factors associated with utilization of newborn postnatal care within two months.

**Results:** This study included 5724 women from the 2006–07 PDHS, 7461 from the 2012–13 survey, and 8287 from the 2017–18 survey. The proportion of women receiving newborn postnatal care within the first two months of delivery increased from 13% in 2006–07 to 43% in 2012–13 but dropped to 27% in 2017–18. Respondent’s occupation and prenatal care utilization of maternal health services were common factors that significantly influenced newborn postnatal care utilization within two months. The utilization of postnatal care was greater among women having educated husbands and where the first child was a male in PDHS 2007 round. Higher wealth index and educated respondent had higher postnatal care utilization odds in DHS 2012 and DHS 2018. However, the odds of using postnatal care decreased with the number of household members and total number of children ever born in DHS 2012 and 2018 rounds.

**Conclusions:** There was a general increase in the proportion of women who utilized postnatal care for their newborns during 2006–2013 but a decrease in 2018. The decreased utilization in 2018 warrants further investigation. Improving women’s economic status, education, employment, and antenatal care attendance and reducing parity may increase newborn postnatal care utilization.

## Introduction

The postnatal period – defined as the first six weeks after birth – is the most critical phase in the lives of mothers and their newborns. Approximately 50% of all maternal and neonatal deaths occur within 24 hours after birth, approximately 60% occur during the first week of life, and the rest occur within six weeks after birth ^1, 2^.

In low and middle income countries, problems such as preterm birth, birth asphyxia, and infections are the leading causes of neonatal deaths ^2^. A striking 99% of the global maternal and neonatal deaths occur in developing countries including Pakistan. Only ten countries, mostly from Asia, account for two-thirds of neonatal deaths. Pakistan reports 7% of global neonatal deaths ^2^ and an estimated 298,000 deaths annually at a mortality rate of 42 per 1000 live births ^3^.

Nowadays, we can save the lives of many newborns through interventions that require only simple technology ^4^. These interventions can be delivered effectively by a skilled birth attendant at home from the first 24 hours of life to 6 weeks ^4^.

 Various policies and health programs ^5^ have been introduced in Pakistan since 1990 to reduce maternal and infant mortality. These include the National Health Policy, National Maternal Newborn and Child Health Program, Pakistan initiative for mothers and newborns, People’s primary healthcare initiative, and Lady Health Worker programs ^5^. However, the effectiveness of a program in improving health indicators depends on the utilization and quality of the services provided. Moreover, reproductive status, family influence, community context, and social and cultural beliefs were found to be significant determinants of postnatal care (PNC) ^6^.

Studies have mentioned that Pakistan does not have a national policy on newborn health, and programs aiming on newborn care are partial in coverage ^7^. Most of the previous studies have focused on assessing the utilization of antenatal or antepartum care services, but only a few have tried to look at postnatal care delivery and utilization. Further most of the studies have focused postnatal care in relation to maternal care, not many studies have foncused of newborn postnatal care. We sought to explore the determinants of newborn PNC utilization over the period from 1991 to 2018 in Pakistan.

## Methods

### Study design and data source

This was a secondary analysis of data from three rounds of Pakistan Demographic and Health Survey (PDHS): PDHS 2006/07, PDHS 2012/13, and PDHS 2017/18. The PDHS 1990–91 did not collect data on postnatal care and hence was excluded from this analysis. The PDHS are nationally representative cross-sectional surveys conducted by the Pakistan Bureau of Statistics with technical support from Opinion Research Corporation (ORC) Macro and funding from US Agency for International Development (USAID). The surveys used a multistage cluster sampling design to collect data on reproductive health, fertility, mortality, family planning, nutrition, and health care utilization. Details about the design of PDHS can be found in published reports ^3^.

This paper was based on previously published data and did not require ethical approval. Permission to use the PDHS datasets was obtained from the DHS Program.

### Study population

The study population comprised women of reproductive age (15–49 years) who gave birth during the last five years preceding the surveys. This included 5725 women from the PDHS 2007/08, 7461 from PDHS 2012/13, and 8287 from the PDHS 2017/18.

### Variables

A conceptual framework proposed by the World Health Organization to explore the social determinants of maternal health was used to consider the various sociodemographic factors that might affect postnatal care utilization. We considered only the variables that were common across the rounds of PDHS.

As there are no nationwide separate newborn health interventions, newborn services are provided with different health programs also considering low postnatal care utilization in Pakistan; the outcome variable constructed was postnatal care of the newborn within two months ^8^. Independent variables were categorized as shown in Box 1.

**Box 1. Categorization of independent variables**

1. Place of residence (urban, rural)

2. Wealth index quintile (poorest, poorer, middle, richer, richest)

3. Number of household members (1–10, 11–20, >20)

4. Number of children younger than five years in household (None, 1–2, 3–4, >4)

5. Births in the last five years (1, 2, >2)

6. Total children ever born (1–2, 3–4, >4)

7. Age of the respondent mother (15–24 years, 25–34 years, 35 years and above)

8. Education status of the respondent mother (No education, primary, secondary, higher)

9. Occupation of the respondent mother (unemployed, employed)

10. Husband’s age (15–24 years, 25–34 years, 35 years and above)

11. Husband’s education (no education, primary, secondary, higher)

12. Husband’s occupation (unemployed, employed)

13. Number of antenatal care (ANC) visits received (no ANC received, <4 visits, ≥4 visits)

### Statistical analysis

Descriptive statistics were used to summarize participants’ characteristics. Because PDHS collected information on postnatal care during the past 5 years, we used the information on the date of birth of the child and receipt of PNC to calculate the coverage of newborn PNC by year for each round of DHS. We used logistic regression models to determine unadjusted and adjusted odds ratios with 95% confidence intervals for the association between the independent variables and newborn PNC. Variables with p <0.1 in the unadjusted (univariable) analysis were included in adjusted (multivariable) analysis. Data were analyzed using SPSS 22.0 software, and P-values < 0.05 were considered to indicate statistical significance.

## Results

### Sociodemographic characteristics of mothers during the three DHS rounds

The mean age of the mothers and her husband was 29.59 and 34 years, respectively, across the three rounds with a little variation as shown in Table 1. The median number of household members was 9, 8, and 8 respectively over the three rounds. However, the number of children ever born was the same in the three rounds. This indicates that the age and family characteristics were nearly the same for Pakistan over the period of 2007–18 as depicted in Table 1.

**Table 1.** Sociodemographic characteristics of respondents from Demographic and Health Surveys (DHS) 2007 to 2018.

Socio-demographic Characteristics	DHS 2007 (n=5724)	DHS 2012 (n=7461)	DHS 2018 (n=8287)
Mean age of respondent (in years)	29.59± 6.8	29.59±6.43	29.56±6.38
Mean age of husband (in years)	34.19±8.56	34.77±7.94	34.56±7.86
Median number of total children (IQR)	1 (5)	1 (4)	1 (4)
Median number of children ever born (IQR)	3 (15)	3 (15)	3 (15)
median number of under five children (IQR)	2 (11)	2 (13)	2 (13)
Median number of household members (IQR)	9 (45)	8(47)	8(47)
Residing in rural locality	3726 (65.1%)	4183 (56.1%)	4549 (54.9%)
Information available on PNC	3625 (63%)	7461 (100%)	8287 (100%)
PNC within 2 months	746 (13%)	3224(43.2%)	2235 (27%)
PNC within 24 hours	374 (6.5%)	2498 (33.5%)	555 (6.7%)

### Factors associated with the utilization of newborn postnatal care

Table 2 shows the results of univariable analysis of factors associated with newborn PNC utilization. The following factors are significantly associated in positive relation in all three surveys: rural residence (p=<0.001), wealth index (p=<0.001), education status of the respondent (p=<0.001), no prenatal care (p=<0.001), and number of antenatal care visits received (p=<0.001). While the number of household members (p=<0.001), number of children aged 5 years or below in the household (p=<0.001), total children ever born (p=<0.001), and births in the last 5 years (p=0.004, p=0.001) were negatively associated with newborn PNC in the 2013 and 2018 surveys.

**Table 2.** Factors affecting newborn postnatal care (PNC) within 2 months in univariable analysis.

Variable	2007 (N=746)		2012 (N=3224)		2018 (N=2235)	
	Unadjusted OR (95% CI)	P value	Unadjusted OR (95% CI)	P value	Unadjusted OR (95% CI)	P value
Rural residence*	0.73 (0.61-0.87)	<0.001	0.75 (0.68-0.82)	<0.001	0.70 (0.63-0.77)	<0.001
**Wealth Index**		<0.001	****	<0.001	****	<0.001
Poorest	Reference		Reference	<0.001	Reference	
Poorer	1.11 (0.89-1.40)	0.36	1.40 (1.20-1.63)	<0.001	1.09 (0.93-1.27)	0.3
Middle	1.23 (0.97-1.56)	0.084	1.64 (1.41-1.92)	<0.001	1.42 (1.22-1.67)	<0.001
Richer	1.62 (1.26-2.07)	<0.001	2.53 (2.18-2.94)	<0.001	1.66 (1.42-1.95)	<0.001
Richest	2.39 (1.78-3.21)	<0.001	5.16 (4.43-6.01)	<0.001	2.55 (2.19-2.98)	<0.001
**Number of household members**		0.432	****	<0.001		<0.001
1 to 10	Reference		Reference	<0.001	Reference	
11 to 20	1.12 (0.94-1.33)	0.216	0.78 (0.71-0.87)	<0.001	0.83 (0.74-0.93)	0.001
More than 20	0.96 (0.64-1.43)	0.830	0.64 (0.49-0.84)	0.001	0.55 (0.39-0.76)	<0.001
**Number of children 5 and under** **in household**		0.478		<0.001		<0.001
No children	Reference		Reference		Reference	
1–2 children	0.72 (0.48-1.10)	0.129	1.02 (0.80-1.31)	0.869	0.80 (0.61-1.04)	0.091
3–4 children	0.71 (0.46-1.10)	0.124	0.82 (0.63-1.07)	0.139	0.67 (0.51-0.88)	0.004
Five or more	0.71 (0.43-1.17)	0.181	0.72 (0.53-0.97)	0.031	0.52 (0.38-0.73)	<0.001
**Births in last five years**		0.413		0.004		0.001
1 child birth	Reference		Reference		Reference	
2 child births	1.11 (0.94-1.32)	0.217	0.92 (0.84-1.02)	0.117	0.83 (0.74-0.92)	<0.001
3 or more child births	0.98 (0.75-1.29)	0.906	0.77 (0.65-0.90)	0.001	0.84 (0.70-1.01)	0.057
**Births in last three years**		0.543		0.334		0.498
No births	Reference		Reference		Reference	
1 child birth	1.11 (0.91-1.36)	0.302	1.08 (0.97-1.21)	0.173	1.05 (0.94-1.18)	0.386
2 or more child births	1.13 (0.87-1.47)	0.365	1.10 (0.94-1.28)	0.225	0.98 (0.84-1.15)	0.817
**Total children ever born**		0.063	****	<0.001	****	<0.001
1–2 children	Reference		Reference	<0.001	Reference	
3–4 children	1.03 (0.84-1.27)	0.763	0.75 (0.67-0.84)	<0.001	0.72 (0.64-0.81)	<0.001
5 or more children	0.83 (0.69-1.01)	0.067	0.54 (0.48-0.60)	<0.001	0.59 (0.52-0.67)	<0.001
**Age of respondent**		<0.001		0.505		0.237
15–24 yrs	Reference		Reference		Reference	
25–34 yrs	0.92 (0.76-1.12)	0.391	0.99 (0.88-1.11)	0.853	1.08 (0.96-1.22)	0.200
35 yrs and above	0.65 (0.51-0.82)	<0.001	0.93 (0.81-1.07)	0.299	0.99 (0.86-1.15)	0.902
**Education status of respondent**		<0.001	****	<0.001	****	<0.001
No education	Reference		Reference		Reference	
Primary	1.58 (1.26-2.00)	<0.001	2.12 (1.85-2.43)	<0.001	1.50 (1.29-1.74)	<0.001
Secondary	2.12 (1.63-2.76)	<0.001	2.39 (2.11-2.71)	<0.001	1.70 (1.50-1.93)	<0.001
Higher	1.97 (1.14-3.41)	0.015	4.29 (3.67-5.00)	<0.001	2.66 (2.32-3.04)	<0.001
**Employed respondent**	1.19 (1.00-1.41)	0.047	1.13 (1.01-1.27)	0.034	1.46 (1.27-1.67)	<0.001
**Husband’s age**		0.001	****	0.009		0.539
15–24 yrs	Reference		Reference		Reference	
25–34 yrs	0.87 (0.65-1.17)	0.362	1.34 (1.11-1.63)	0.003	1.10 (0.91-1.35)	0.329
35 yrs and above	0.67 (0.50-0.89)	0.006	1.25 (1.03-1.52)	0.024	1.06 (0.87-1.29)	0.559
**Husband’s education status**	****	<0.001	****	<0.001		<0.001
No education	Reference		Reference	<0.001	Reference	
Primary	1.59 (1.26-1.99)	<0.001	1.60 (1.37-1.86)	<0.001	1.23 (1.04-1.45)	0.017
Secondary	1.56 (1.28-1.90)	<0.001	1.71 (1.52-1.93)	<0.001	1.39 (1.23-1.59)	<0.001
Higher	2.30 (1.77-2.98)	<0.001	2.75 (2.41-3.13)	<0.001	1.69 (1.47-1.94)	<0.001
**Employed Husband**			1.84 (1.35-2.50)	<0.001		
**No Pre-natal care**	2.98 (2.50-3.55)	<0.001	3.74 (3.31-4.22)	<0.001	0.32 (0.27-0.38)	<0.001
**Number of ANC visits received**	****	<0.001	****	<0.001	****	<0.001
No ANC received	Reference		Reference	<0.001	Reference	
Less than 4 visits	2.37 (1.97-2.84)	<0.001	1.92 (1.70-2.17)	<0.001	2.12 (1.76-2.57)	<0.001
4 or more visits	3.90 (3.09-4.93)	<0.001	4.09 (3.62-4.63)	<0.001	3.91 (3.27-4.68)	<0.001
**Male sex of previous child**	1.29 (1.10-1.52)	0.002	0.96 (0.88-1.05)	0.391	1.12 (1.02-1.23)	0.023

OR – odds ratio

Regression analysis was performed for the significant independent variables found in each dataset to control for confounding and derive adjusted odds ratio ( Table 3). Multivariable logistic regression analysis unadjusted results are shown in Table 3. In the 2007 survey, occupation of the respondent (p=0.015, OR=1.26), husband's education (p=0.006), prenatal care utilization (p=<0.001 OR= 3.57), sex of the previous child (p=0.002, OR= 1.30), and number of antenatal visits (p=0.001) were significantly associated with newborn PNC within two months.

**Table 3.** Factors affecting newborn postnatal care (PNC) within two months in multivariable analysis.

Variable	2007 (N=746)		2012 (N=3224)		2018 (N=2235)	
	Adjusted OR (95% CI)	P value	Adjusted OR (95% CI)	P value	Adjusted OR (95% CI)	P value
Rural residence*	0.99 (0.81-1.23)	0.984	1.68 (1.49-1.90)	<0.001	0.98 (0.87-1.10)	0.717
**Wealth Index**		0.718		<0.001	****	0.001
Poorest	Reference		Reference		Reference	
Poorer	0.94 (0.74-1.19)	0.604	1.29 (1.09-1.52)	0.003	0.93 (0.79-1.10)	0.415
Middle	0.91 (0.69-1.19)	0.492	1.41 (1.18-1.68)	<0.001	1.03 (0.86-1.24)	0.757
Richer	1.01 (0.75-1.37)	0.939	2.05 (1.69-2.49)	<0.001	1.05 (0.86-1.29)	0.626
Richest	1.15 (0.78-1.69)	0.489	3.67 (2.93-4.59)	<0.001	1.39 (1.11-1.74)	0.004
**Number of household members**		NS		0.042		0.306
1 to 10			Reference		Reference	
11 to 20			0.85 (0.75-0.97)	0.015	0.93 (0.81-1.07)	0.307
More than 20			0.78 (0.56-1.11)	0.167	0.76 (0.51-1.12)	0.166
**Number of children 5 and under** **in household**		NS		0.491		0.165
No children			Reference		Reference	
1–2 children			1.15 (0.88-1.50)	0.323	0.92 (0.70-1.21)	0.554
3–4 children			1.07 (0.80-1.44)	0.630	0.82 (0.61-1.10)	0.19
Five or more			0.98 (0.68-1.41)	0.905	0.71 (0.48-1.03)	0.071
**Births in last five years**		NS		0.233		0.079
1 child birth			Reference		Reference	
2 child births			1.09 (0.98-1.22)	0.122	0.94 (0.84-1.05)	0.287
3 or more child births			1.13 (0.93-1.38)	0.229	1.19 (0.96-1.48)	0.111
**Births in last three years**		NS		NS		NS
**Total children ever born**		NS		0.001		<0.001
1–2 children			Reference		Reference	
3–4 children			0.79 (0.69-0.91)	0.001	0.79 (0.70-0.89)	<0.001
5 or more children			0.78 (0.67-0.91)	0.002	0.79 (0.69-0.91)	0.001
**Age of respondent**		0.436		NS		NS
15–24 yrs	Reference					
25–34 yrs	1.03 (0.81-1.30)	0.839				
35 yrs and above	0.88 (0.64-1.21)	0.422				
**Education status of respondent**		0.266		<0.001		<0.001
No education	Reference		Reference		Reference	
Primary	1.18 (0.91-1.52)	0.211	1.38 (1.19-1.61)	<0.001	1.17 (0.99-1.38)	0.059
Secondary	1.33 (0.97-1.81)	0.074	1.17 (0.99-1.37)	0.052	1.15 (0.99-1.34)	0.076
Higher	1.04 (0.57-1.89)	0.908	1.54 (1.25-1.90)	<0.001	1.53 (1.26-1.84)	<0.001
**Employed respondent**	1.26 (1.05-1.51)	0.015	1.43 (1.26-1.62)	<0.001	1.45 (1.26-1.68)	<0.001
**Husband’s age**		0.192		0.026		NS
15–24 yrs	Reference		Reference			NS
25–34 yrs	0.77 (0.55-1.07)	0.118	1.21 (0.98-1.49)	0.075		
35 yrs and above	0.71 (0.49-1.03)	0.070	1.35 (1.08-1.69)	0.010		
**Husband’s education status**		0.006		0.034		0.189
No education	Reference		Reference		Reference	
Primary	1.37 (1.07-1.74)	0.011	1.24 (1.06-1.47)	0.010	0.98 (0.82-1.17)	0.846
Secondary	1.24 (0.99-1.55)	0.062	1.01 (0.88-1.16)	0.885	0.95 (0.82-1.10)	0.479
Higher	1.62 (1.19-2.19)	0.002	1.10 (0.92-1.31)	0.264	0.84 (0.70-1.00)	0.046
**Employed Husband**			1.61 (1.16-2.24)	0.004		
**No Pre-natal care**	3.57 (2.13-5.98)	<0.001	2.74 (2.08-3.61)	<0.001	0.57 (0.22-1.43)	0.229
**Number of ANC visits received**		0.001		<0.001		<0.001
No ANC received	Reference		Reference		Reference	
Less than 4 visits	0.65 (0.39-1.09)	0.100	0.76 (0.59-0.98)	0.031	1.12 (0.45-2.79)	0.816
4 or more visits	1.01 (0.59-1.71)	0.986	1.18 (0.92-1.51)	0.199	1.67 (0.67-4.18)	0.271
**Male sex of previous child**	1.30 (1.09-1.54)	0.002			1.14 (1.03-1.26)	0.011

OR – odds ratio, NS - Not significant in univariable analysis; not included in the multivariable analysis

In the 2012 survey, the same factors as those of the 2007 survey, namely, occupation of the respondent (p=<0.00, OR= 1.43), husband's education (p=0.034), prenatal care utilization (p=<0.001, OR= 2.74), and number of antenatal visits (p=<0.001), also showed significantly positive associations. In addition, factors such as wealth index (p=<0.001), education of the respondent (p=<0.001), and husband occupation (p=0.004, OR= 1.61) also showed positive associations. However, the number of household members (p=0.042) and total children ever born (p=0.001) were negatively associated with newborn PNC utilization.

In the 2018 survey, receiving postnatal care within two months of birth was significantly associated with the occupation of the respondent (p=<0.001, OR=1.45), education of the respondent (p=<0.001), wealth index (p=0.001), number of antenatal visits (p=<0.001), and sex of the first child (p=<0.011, OR=1.14). However, women who had more total children ever born (p=<0.001) were less likely to receive newborn care utilization.

## Discussion

The current study aimed to explore newborn postnatal care determinants from three subsequent rounds of DHS 2006–07, 2012–13, and 2017–18 in Pakistan. Various sociodemographic factors along with household characteristics and utilization of antenatal care services determine the utilization of PNC, in general, from past literature ^9, 10^. We also extracted relevant data from three rounds of PDHS pertaining to potential factors, which could affect the utilization of newborn postnatal care in this study as described in the methods section.

The number of respondents for the three rounds of PDHS was 5724, 7461, and 8287, respectively, for the years 2007, 2013, and 2018, respectively. It was found that the utilization of PNC for mothers and newborn within two months following delivery increased from 13% to 43% in 2013 and the subsequently reduced to 27% in 2018. Similarly, the utilization of PNC within 24 hours increased from 7% in 2007 to 33% in 2013 and reduced to 7% in 2018 ( Table 1). This non-linear pattern in service utilization could be due to distinct geographical regions in which the survey was carried out. During DHS 2006–07, data were collected from four regions: Punjab, Sindh, Khyber Pakhtunkhwa (KPK), and Balochistan. In the next round of DHS 2012–13, along with Punjab, Sindh, and Balochistan; three other districts of KPK, Gilgit Baltistan (GB), and Islamabad were included. Similarly, in DHS 2017–18, seven regions, namely, Balochistan, Punjab, Sindh, KPK, GB, Azad Jamu and Kashmir, Islamabad Capital Territory and Fata constituted the sampling frame. The sociodemographic characteristics along with the distribution of health services and quality would have been different, which may have resulted in varied PNC utilization levels across sample ^11^. The study conducted by Iqbal S *et al.* also indicated variability in PNC service utilization across different regions from where the data were collected ^10^.

Among all the sociodemographic determinants included in this study, the occupation of the respondent and the utilization of ANC ( Table 4) were found to be significantly associated with newborn PNC utilization within two months after delivery across all the three rounds of DHS. It was found that the odds of using PNC was 1.26 times more among women who were employed than among unemployed mothers. Previous studies conducted in Pakistan ^10^ and from other neighboring countries also showed a positive association among mothers with employment ^12– 14^ ( Table 3). However, wealth index of household ^15, 16^, education status of the respondents ^15, 16^, and total children ever born ^16, 17^ were significantly associated with newborn PNC for two DHS rounds: 2012–13 and 2017–18. Utilization of maternal health services, especially antenatal or prenatal care, was also a strong predictor of PNC throughout all PDHS; it is evident from the literature that ANC is the entry point for the utilization of maternal health services during and after pregnancy ^18– 21^. The respondent’s occupation and utilization of antenatal care were found to be associated with newborn PNC from the DHS 2006–07 and 2012–13 data in previous studies ^8, 10^.

**Table 4.** Significant factors influencing newborn postnatal care (PNC) utilization within two months.

2007	2012	2018
Significant factors for new-born PNC utilization within two months		
Occupation of respondent	Occupation of respondent	Occupation of respondent
Husband's education	Husband's education	Education of respondent
Prenatal care utilization	Wealth index	Wealth index
Sex of previous child	Education of respondent	Total children ever born
No. of antenatal visits	Husband's age	No. of antenatal visits
	Husband's occupation	Sex of first child
	Number of household members	
	Total children ever born	
	Prenatal care utilization	
	No. of antenatal visits	

This study indicates that the occupation of the respondent and prenatal care services utilization by respondents influenced the utilization of newborn PNC across all the three rounds of the PDHS. Other common factors such as wealth index, education of the respondent, and total number of children ever born also influenced the uptake of newborn PNC services. Another strength of this study is the number of sociodemographic and outcome variables included, which is far higher than those included in previous studies ^8, 10^.

However, we could not see determinants of newborn PNC utilization due to data unavailability on PNC from the 1990–91 PDHS. Moreover, the data on the reasons for not getting the PNC by the women after delivery were not available.

There are limitations to the data, which we noticed while conducting this analysis. These limitations may be considered as recommendations for further improving the scope of the DHS. There was no information available on the distribution of health services in the DHS data. This information is important, as differential health service availability and accessibility directly influence PNC utilization, which we could not explore in the current study. In future research, the data could be used to link the availability and accessibility of services with their utilization. The second limitation in data, we noticed, was regarding the quality of PNC, which was not captured in the DHS questionnaire. This question is crucial to explain the reducing uptake of newborn PNC services, especially in 2017–18. The DHS also did not contain any information on the domains for which PNC is provided, which is again important for improving the health of the mother and the newborn.

## Conclusions

This study reveals that women being employed, utilization of ANC or prenatal services, wealth index, and education of respondents or their husbands increases the uptake of newborn PNC utilization. An increasing number of children ever born to women are less likely to have newborn PNC utilization. Hence, there is a need to address the issues of improving economic status, education, employment of the women, and population control to increase newborn PNC utilization. Similarly, interventions that increase the coverage and quality of ANC services will also increase the utilization of newborn PNC among women in Pakistan.

## Data availability

### Source data

The data for this study is owned by the DHS Program. The Individual Recode datasets for the PDHS 2006–07, 2012–13 and 2017–2018 were used for this study and can be obtained here: https://www.dhsprogram.com/data/available-datasets.cfm?ctryid=31

The electronic data is available from the DHS Program under its terms of use. Before downloading the data, users must register as a DHS user for reasons laid out on the DHS Program website and dataset access is only granted for legitimate research purposes.
