# Supplementary material for: Determinants of newborn care utilization in Pakistan: Findings from the Demographic and Health Surveys
Source: F1000Res. 2020 Oct 21;9:1061. Originally published 2020 Aug 28. [Version 2] doi: 10.12688/f1000research.25700.2 (PMC7658725; doi:10.12688/f1000research.25700.2)
Supplement: Peer review history

# Peer review: Reviewer response for version 2

**DOI:** 10.5256/f1000research.30160.r73446

**Published:** 2020-11-11

**Authors:** Korravarn Yodmai

**Affiliations:** 1 Department of Family Health, Faculty of Public Health, Mahidol University, Bangkok, Thailand

No more comment and question in this article.

Is the work clearly and accurately presented and does it cite the current literature?

Partly

If applicable, is the statistical analysis and its interpretation appropriate?

Partly

Are all the source data underlying the results available to ensure full reproducibility?

Yes

Is the study design appropriate and is the work technically sound?

Yes

Are the conclusions drawn adequately supported by the results?

Yes

Are sufficient details of methods and analysis provided to allow replication by others?

Partly

Reviewer Expertise:

reproductive health

I confirm that I have read this submission and believe that I have an appropriate level of expertise to confirm that it is of an acceptable scientific standard.

# Peer review: Reviewer response for version 1

**DOI:** 10.5256/f1000research.28364.r70513

**Published:** 2020-10-07

**Authors:** Ejaz Ahmad Khan

**Affiliations:** 1 Department of Epidemiology and Biostatistics, Health Services Academy (HSA), Islamabad, Pakistan

1. Title of the manuscript need to aligned with the objective of the study i.e., post natal care and NOT the postpartum case.
2. Abstract needs to be rewritten in proper academic English with flow.
3. Authors leave the conclusion with a question about 2018 data, which they should have had discussed in their discussion section before concluding their remarks.
4. Introduction: Pakistan is a Low-middle income country and NOT a low income country as per the World Bank ranking. Introduction needs more robust literature cited. 
5. Methods need to be in the past tense and so should be the results.
6. Results need to be rewritten in a proper sequence and flow. The DHSs analysed year-wise need to be written together for each background factor. 
7. Discussion must discuss the most important results as per the objectives of the study, and critique 2018's unexpected results with good literature support. 

Is the work clearly and accurately presented and does it cite the current literature?

Partly

If applicable, is the statistical analysis and its interpretation appropriate?

Yes

Are all the source data underlying the results available to ensure full reproducibility?

Yes

Is the study design appropriate and is the work technically sound?

Yes

Are the conclusions drawn adequately supported by the results?

Partly

Are sufficient details of methods and analysis provided to allow replication by others?

Yes

Reviewer Expertise:

Systematic reviews, epidemiology, burden of disease

I confirm that I have read this submission and believe that I have an appropriate level of expertise to confirm that it is of an acceptable scientific standard.

# Peer review: Reviewer response for version 1

**DOI:** 10.5256/f1000research.28364.r70514

**Published:** 2020-09-10

**Authors:** Korravarn Yodmai

**Affiliations:** 1 Department of Family Health, Faculty of Public Health, Mahidol University, Bangkok, Thailand

1. In a part of the introduction,  the gap of knowledge based on current studies should be identified. 
2. Some details of methods such as target population, where was data collected such as there was collected data from community or health facility. It may important to present the data collecting process (briefly). 
3. Interpretation of the result should explain more detail the magnitude of the result rather than explain just the association. 
4. In the discussion, the authors should explain more detail about PNC services in each area. What is a barrier of service in those areas, even they may use the same health policy or need to succeed in the same indicators? 
5. According to the introduction part, this study has filled the gap of knowledge or not.      

Is the work clearly and accurately presented and does it cite the current literature?

Partly

If applicable, is the statistical analysis and its interpretation appropriate?

Partly

Are all the source data underlying the results available to ensure full reproducibility?

Yes

Is the study design appropriate and is the work technically sound?

Yes

Are the conclusions drawn adequately supported by the results?

Yes

Are sufficient details of methods and analysis provided to allow replication by others?

Partly

Reviewer Expertise:

reproductive health

I confirm that I have read this submission and believe that I have an appropriate level of expertise to confirm that it is of an acceptable scientific standard, however I have significant reservations, as outlined above.

# Response

**Published:** 2020-10-15

**Authors:** ABDUL GHAFFAR

**Affiliations:** Chulalongkorn University, Pakistan

In response to comment 1, from Korravarn Yodmai

Knowledge gap added

In response to comment no 2 from Korravarn Yodmai,

DHS has standard protocols and data method process is already briefly mentioned, for more details reader are referred to reference below.

In response to comment 3, from Korravarn Yodmai

More details added, OR added in results description

In response to comment 4, from Korravarn Yodmai

These data represent entire Pakistan and barrier in different provinces may not be possible to consider in discussion section as we don’t have data about barriers in DHS surveys. Details about PNC services are provided in the introduction and were not included in discussion section due to repetition, and also it is mentioned that currently there is no separate newborn health policy in Pakistan

Response to comment 5 from Korravarn Yodmai

Yes the study fulfilled the knowledge gap, as conclusion describes the findings which are consistent in all three surveys like education of women and utilization of prenatal services
